# Evaluating the ability of the pairwise joint site frequency spectrum to co-estimate selection and demography

**DOI:** 10.3389/fgene.2015.00268

**Published:** 2015-08-17

**Authors:** Lisha A. Mathew, Jeffrey D. Jensen

**Affiliations:** School of Life Sciences, École Polytechnique Fédérale de LausanneLausanne, Switzerland

**Keywords:** joint site frequency spectrum, joint estimation, selection and demography, genetic hitchhiking, positive selection

## Abstract

The ability to infer the parameters of positive selection from genomic data has many important implications, from identifying drug-resistance mutations in viruses to increasing crop yield by genetically integrating favorable alleles. Although it has been well-described that selection and demography may result in similar patterns of diversity, the ability to jointly estimate these two processes has remained elusive. Here, we use simulation to explore the utility of the joint site frequency spectrum to estimate selection and demography simultaneously, including developing an extension of the previously proposed Jaatha program (Mathew et al., 2013). We evaluate both complete and incomplete selective sweeps under an isolation-with-migration model with and without population size change (both population growth and bottlenecks). Results suggest that while it may not be possible to precisely estimate the strength of selection, it is possible to infer the presence of selection while estimating accurate demographic parameters. We further demonstrate that the common assumption of selective neutrality when estimating demographic models may lead to severe biases. Finally, we apply the approach we have developed to better characterize the within-host demographic and selective history of human cytomegalovirus (HCMV) infection using published next generation sequencing data.

## Introduction

Identifying the action of selection using genomic polymorphism data has been a long sought after goal of population genetics, and several computational methods have been proposed. One of the most widely used is that of Kim and Stephan (2002), who utilized a composite-likelihood-ratio to empirically test models of neutrality against positive selection, a framework on which a number of subsequent methods have been built (e.g., Kim and Nielsen, 2004; Jensen et al., 2005). These approaches assume that the population is at equilibrium, and thus forgo any understanding of the underlying demographic history of the population. A related class of methods instead employs a background site frequency spectrum within this same likelihood framework in an attempt to account for the underlying and unknown demographic history (e.g., Nielsen et al., 2005; Pavlidis et al., 2010).

While it has long been appreciated that demographic perturbations (e.g., population size change, population structure, migration) may result in patterns of variation that are similar to those produced under positive selection, and should therefore be taken into account when identifying selected regions (e.g., Robertson, 1975; Andolfatto and Przeworski, 2000; Teshima et al., 2006; Thornton and Jensen, 2007; Siol et al., 2010; Jensen, 2014), it has also been specifically demonstrated that the assumption of an equilibrium population history may bias selection inference (e.g., Jensen et al., 2005). Further, Crisci et al. (2013) recently evaluated several proposed background site frequency spectrum based approaches [including Sweepfinder (Nielsen et al., 2005), Sweed (Pavlidis et al., 2013), OmegaPlus (Alachiotis et al., 2012), and iHS (Voight et al., 2006)]. Though they demonstrated the linkage disequilibrium based approaches to perform better, they also described a high false positive rate and low true positive rate under a great variety of models – most notably those including severe bottlenecks.

The most common approach in the field currently is to first infer demography using assumed neutral sites (e.g., synonymous or intergenic sites), and then use this inference to search for regions that have unusual patterns compared to the background (e.g., Nielsen et al., 2005), with the notion that these outliers may have recently been shaped by the action of positive selection (Williamson et al., 2005). Apart from the concerning simulation results of Crisci et al. (2013) discussed above, there is accumulating evidence that these assumed-neutral classes of sites may be impacted by selection either directly or via linkage (e.g., DuMont et al., 2004; Gartner et al., 2013; Hietpas et al., 2013; Bank et al., 2014a). Further, the assumption of a purely neutral class of sites unaffected by direct or linked selection is tenuous in many populations of interest (see Zhou et al., 2014). These combined results thus suggest that the best direction forward is the development of novel approaches to infer selection and demography simultaneously, particularly given that adaptive events are often associated with demographic changes, as in the colonization of novel habitats (e.g., Domingues et al., 2012; Poh et al., 2014). To date, however, there is no program available for simultaneous co-estimation.

Here we explore the utility of the joint site frequency spectrum (JSFS) for the simultaneous estimation of demographic parameters and selection strengths under a hitchhiking model in non-equilibrium populations. The JSFS is an *n*-dimensional array that counts the numbers of derived polymorphisms in different mutational classes of the *n* populations under consideration. In our simulation study we consider the JSFS *J* of two populations *P_1_* and *P_2_* (e.g., *J [x, y]* = *z* means that there are *z* positions in our aligned data that are found in *x* samples of in *P_1_* and in *y* samples in *P_2_*). As already extensively tested in Tellier et al. (2011), we further coarsen the JSFS into Jaatha’s default set of summary statistics (SS) based upon frequency classes (for a description see Naduvilezhath et al., 2011; Tellier et al., 2011; Mathew et al., 2013; and an example in Supplementary Figure S1), which has been shown to perform well when estimating neutral demographic models. These SS divide the high and low frequency variants into single frequency classes and the middle frequency variants into fewer classes, resulting in 23 frequency classes in total. For example, in Supplementary Figure S1 for a sample size of 15 and beginning in the upper left corner of the JSFS, the first SS consists of sites fixed (frequency 15/15) in population 1 and absent (frequency 0/15) in population 2, the second SS consists of sites fixed in population 1 and at frequency 1/15 or 2/15 in population 2, the third SS consists of sites fixed in population 1 and at a frequency between 3/15 and 12/15 in population 2, and so on. These 23 SS are treated as a vector of length 23 with each element being equally weighted by 1.

For the purpose of making this simulation study tractable, we focus on the specific scenario in which positively selected alleles are introduced in a single individual in the selected population at the time of a population split, as one might expect when a population colonizes a novel habitat. We consider two models: an ongoing sweep and a completed sweep. We utilize the simulation program *msms* (Ewing and Hermisson, 2010) with a modification of Jaatha 2.0 in the case of the complete sweep scenario. The simulator *msms* first simulates forward in time the trajectory of the selected allele and then, conditioned on that trajectory, simulates the demographic events backward in time. To investigate the inference accuracy of demographic (population sizes, divergence time, migration rate, size changes) together with selection parameters, we use the composite-likelihood method Jaatha 2.0, which has been demonstrated to give accurate estimates for neutral cases (Mathew et al., 2013). Of primary interest is determining if the information contained within the JSFS suffices to estimate both demographic and selective parameters simultaneously. Under the chosen scenarios, including a coinciding size change during the selection process, we are able to recover the demographic parameters accurately and to distinguish between purely neutral demographic histories and those incorporating selection. In agreement with the results of Crisci et al. (2013), we find that incorrectly assuming neutrality results in severe biases after a complete sweep.

Finally, as an application of this developed approach, we investigate the selective and demographic history of human cytomegalovirus (HCMV), a common β-herpes virus with seroprevalence of 30–90% in the USA (Dowd et al., 2009). This population was chosen for study as the demographic history associated with infection has been investigated extensively and described to include drastic population size changes (see Renzette et al., 2011, 2013, 2014); strong evidence of extensive positive selection associated with colonization has also been described – impacting ∼20% of open reading frames across the genome (Renzette et al., 2013). Thus, this population represents an ideal test case in which both factors are thought to be strongly at play during the colonization (i.e., infection) of a new individual.

## Materials and Methods

### Simulating Demography

**FIGURE 1:**
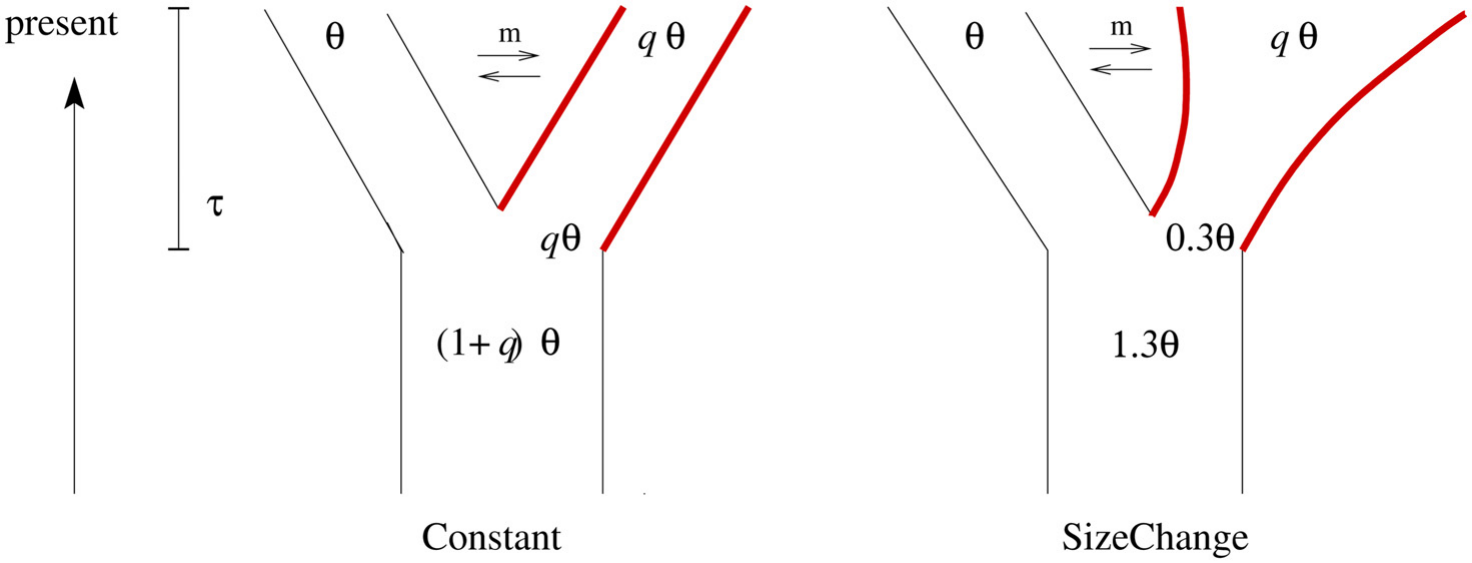
**In both demographic models used for the simulation study – *Constant* and *SizeChange* – one ancestral population splits into two daughter populations at time τ followed by continuous, bidirectional gene flow**. Selection is simulated by introducing a single selected site at the time of the split in the second population only (marked in red; for more details see text).

We investigate two models – an ongoing sweep and a completed sweep – under two demographic models involving a population split (**Figure 1**). In both models, population *P_1_* (with θ = 4*N_e_*μ with *N_e_* being the effective population size of *P_1_*) stays constant in size after the split from the ancestral population. The ancestral population of size (1+*u*)*N_e_* splits at τ generations (measured in units of 4*N_e_*) before the present into two populations. In the *Constant* model *P_2_* stays unchanged in size after the split (i.e., *u* = *q*). In the *SizeChange* model *P_2_* exponentially changes its size from *uN_e_* to *qN_e_* and *u* = 0.3; thus if *q* > 0.3, *P_2_* increases in size following the split. Migration is assumed to be symmetric between *P_1_* and *P_2_* and is also measured in units of 4*N_e_*, as in *msms* (Ewing and Hermisson, 2010).

### Simulating Selection

Positively selected alleles arise at the time of the split with a frequency of 1/(*uNe)* in *P_2_* only and experiences a selection strength of α = 2*N_e_s* during the time following the split. In both scenarios we condition on the selected allele being present not only in the population but also in the sample; further, the selected allele is assumed to be in the center of each locus. The average frequency of the selected allele *f* per locus in each data set for the incomplete sweep scenario ranges from 0.017 to 0.96. For the scenario of the completed sweep, we modified Jaatha 2.0 in order to condition on fixation of the selected allele.

### Parameter Inference

The joint estimation of selection and demography was performed with Jaatha 2.0 with *msms* as an internal simulator under the specified models. To quantify the impact of incorrectly assuming a neutral model in both scenarios, we also ran neutral estimations under the *SizeChange* model. For the incomplete and the complete sweep scenarios, we investigated the accuracy with 20 and 14 simulated data sets, respectively, each consisting of 100 loci of 1 kb length under the infinite-sites model, with 25 samples from each population; both scenarios were tested using both the *Constant* and the *SizeChange* model. In the datasets, each sampled locus represents either the generation of random genomic regions for the purposes of demographic estimation or the further evaluation of previously identified regions containing putatively beneficial mutations. This represents a simplistic approach in which beneficial mutations are occurring at an underlying rate across the genome (though we do not here attempt to separately estimate a beneficial mutation rate, as the beneficial mutations are here modeled to arise in response to the colonization of a novel habitat at the time of the split from the ancestral population, and not at a constant rate as is commonly assumed under recurrent hitchhiking models). We also used an incomplete sweep model analysis on data sets generated under a complete sweep model in order to investigate the effects of model mis-specification. The chosen parameter ranges, *msms* commands (*msms* version 3.2rc Build:74), and Jaatha settings are given in the Supplement A1. Plotted are the results with the highest likelihood.

### Data Application

We have utilized the HCMV next-generation-sequencing-data set from the 6-months collection of urine and plasma from patient B103 described in Renzette et al. (2013). Based on the branch statistic (PBS) results of Renzette et al. (2013) we defined a region containing a significant PBS statistic in coding regions such that the largest possible region of an open reading frame was selected with the identified selected site in the center. This procedure yielded an average region size of 765 bp. Three putatively selected sites outside of ORFs were identified by Renzette et al. (2013). Keeping those sites centered, regions of 765 bp on either side were included. The resulting dataset included 15 putatively selected regions (8960 SNPs, each region of ∼1000 bp) for which joint estimation was performed with a neutral and a selection model (see Supplementary Figure S2 for the JSFS of the HCMV data).

Based on the neutral demographic estimations of Renzette et al. (2013) we developed a demographic model in which an ancestral population undergoes a bottleneck representing initial infection, and subsequently undergoes a second bottleneck in to the urine population at the time of the split of the plasma and urine populations. In the plasma population, selection start time is modeled variably between the time of split and the present. We assume constant size in the plasma population and model selection in this population only. We also assume that the urine population increases in size after the split and that there is no migration between the two compartments. The sample size is set to 15, which corresponds to the minimal coverage of sites that pass our quality control. Sites that were sampled with a higher coverage were scaled down proportionally.

## Results

**FIGURE 2:**
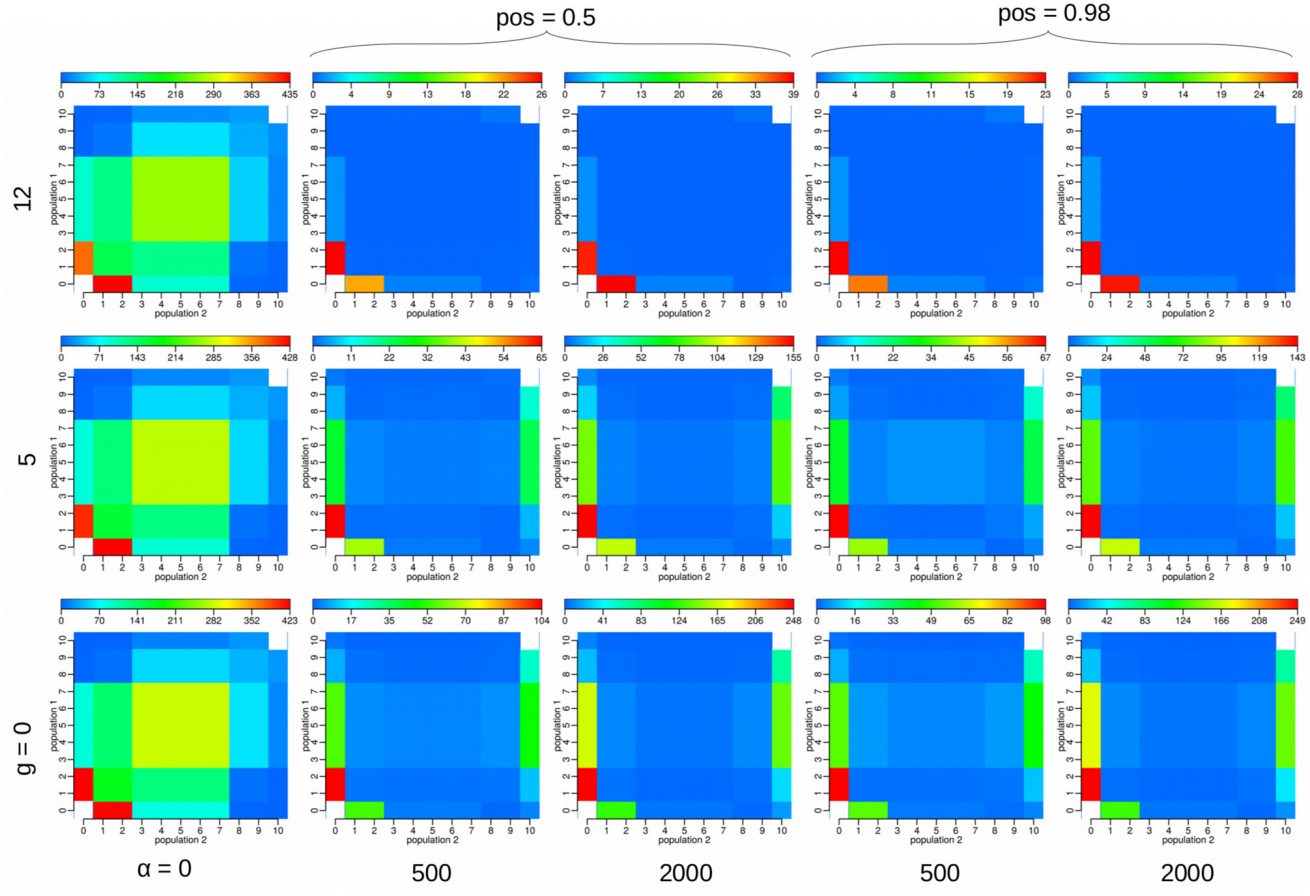
**We visualized the average values of the 23 summary statistics (SS) used with three differing parameters under the *SizeChange* model under a complete sweep scenario: selection strength α = 2*N_e_s*, the exponential growth rate *g* of the second population in which also the selected allele arises, and the position of the selected allele *pos***. For each plot we ran 100 replicates of 100 loci each of 1 kb in length, with 10 samples from each population. For example, the field [2,3] in the matrix represent the number of SNPs found in exactly two samples in population 1 and three samples in population 2. The other simulation parameters were fixed to the following values: τ = 0.05, *N_e_* = 1000, θ_site_ = 0.004, *m* = 0.2, and recombination rate per site = 1.64⋅10^-4^. When comparing, note that the scale (placed above each JSFS plot) is different for each subfigure. The distinction between the neutral and selected cases is clearly visible, owing to the decrease in polymorphisms in the selected case. The higher the growth rate *g* becomes, however, the closer in number and more similar the SS values. The SS show only very minute differences owing to differing locations of the selected allele.

**FIGURE 3:**
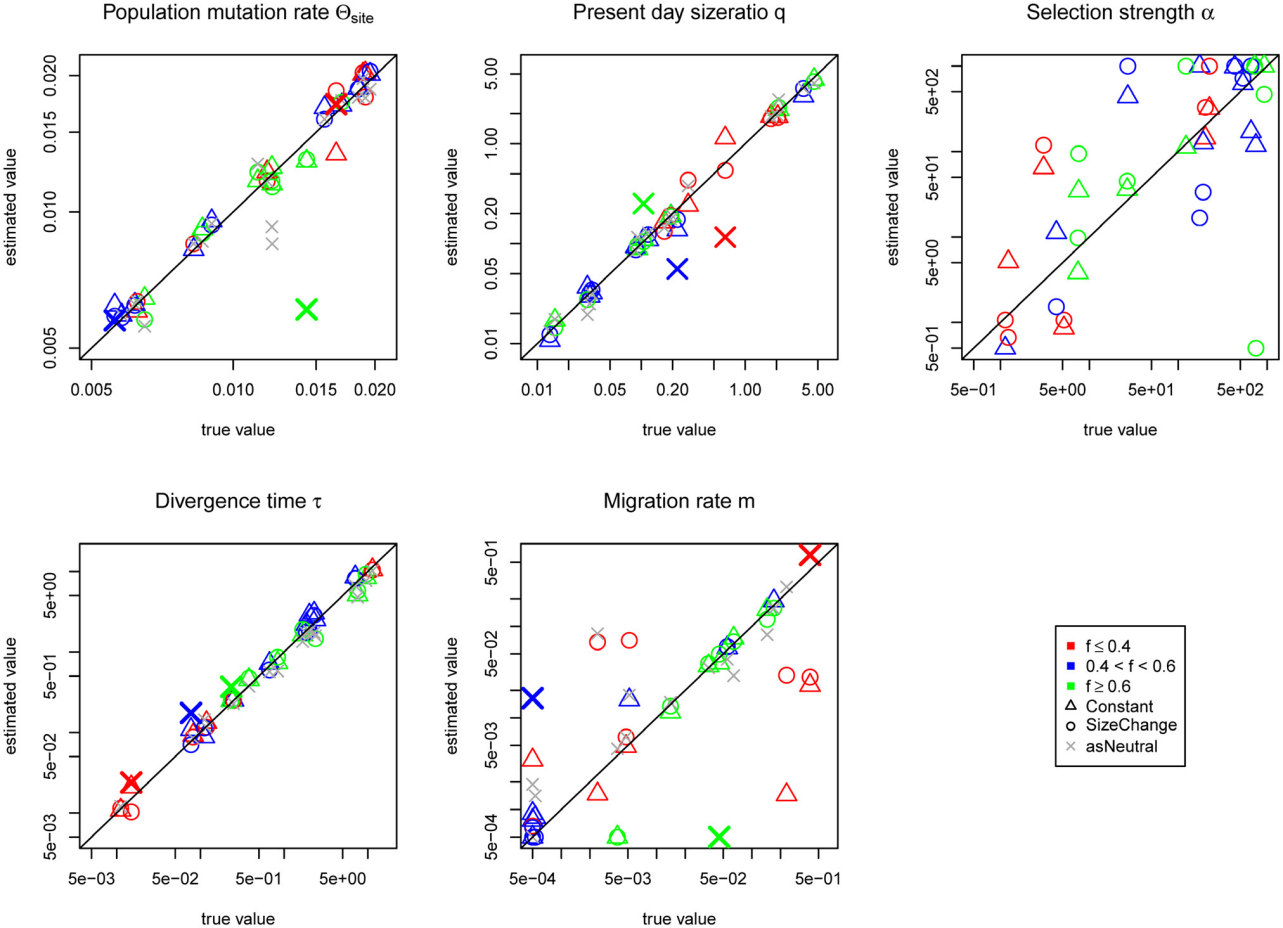
**Jaatha results of the simulation study under the incomplete sweep scenario for the *Constant* (Δ) and the *SizeChange* (o) model**. For the *SizeChange* model neutral estimates are also given (x, as Neutral). The average frequency of the selected allele *f* in the data set is colored accordingly. To distinguish which estimates came from the same data set, three neutral estimates are color-coded and increased in size and show the covariance of the present day population size *q* with the migration rate *m*. These three colored ‘X’s represent the neutral estimate for the corresponding colored *SizeChange* (o) estimates. The results are plotted in the parameter ranges that were evaluated in Jaatha. The true values of the data sets produced with no migration were set manually to the lowest value of the parameter range. The demographic parameters are estimated accurately, however, α is not. The estimation of *m* improves with increasing frequency of the selected allele in the data sets. Except for a few cases, if one incorrectly assumes neutrality the demographic parameters are still correctly recovered, as shown by the fact that they often lie on or near the diagonal.

Under the *SizeChange* model we performed simulations with *msms* (Ewing and Hermisson, 2010) and visualized the chosen SS. The SS between the neutral and selected cases appear distinguishable, both in frequency distribution and number (**Figure 2**, Supplementary Figure S3). However, changing the selection strength did not significantly alter observed patterns owing to the region size; this suggests the ability to only reject neutrality, rather than to estimate precise selective parameters. With increasing growth rates of *P_2_*, the population in which the selected allele arose, the SS and the number of polymorphisms produced with differing strengths of selection become increasingly difficult to discriminate. However, increasing the size of the locus improves the ability to distinguish between differing strengths of selection (cp. Supplementary Figure S3 and **Figure 2**), as expected, owing to the ability to characterize the size of the hitchhiked region (see Jensen et al., 2008). Under the incomplete sweep scenario we find that the demographic parameters can be accurately estimated, but not the selection strength α (**Figure 3**), though low and high α values appear to be distinguishable. The average frequency *f* of the selected allele is important for the accuracy of the estimation of the migration rate *m*. The lower *f* is the more difficult the estimation of the migration rates becomes. Although we simulated data under selection we found no obvious impact of incorrectly assuming a neutral model under the incomplete sweep scenario (except for an underestimation of θ).

**FIGURE 4:**
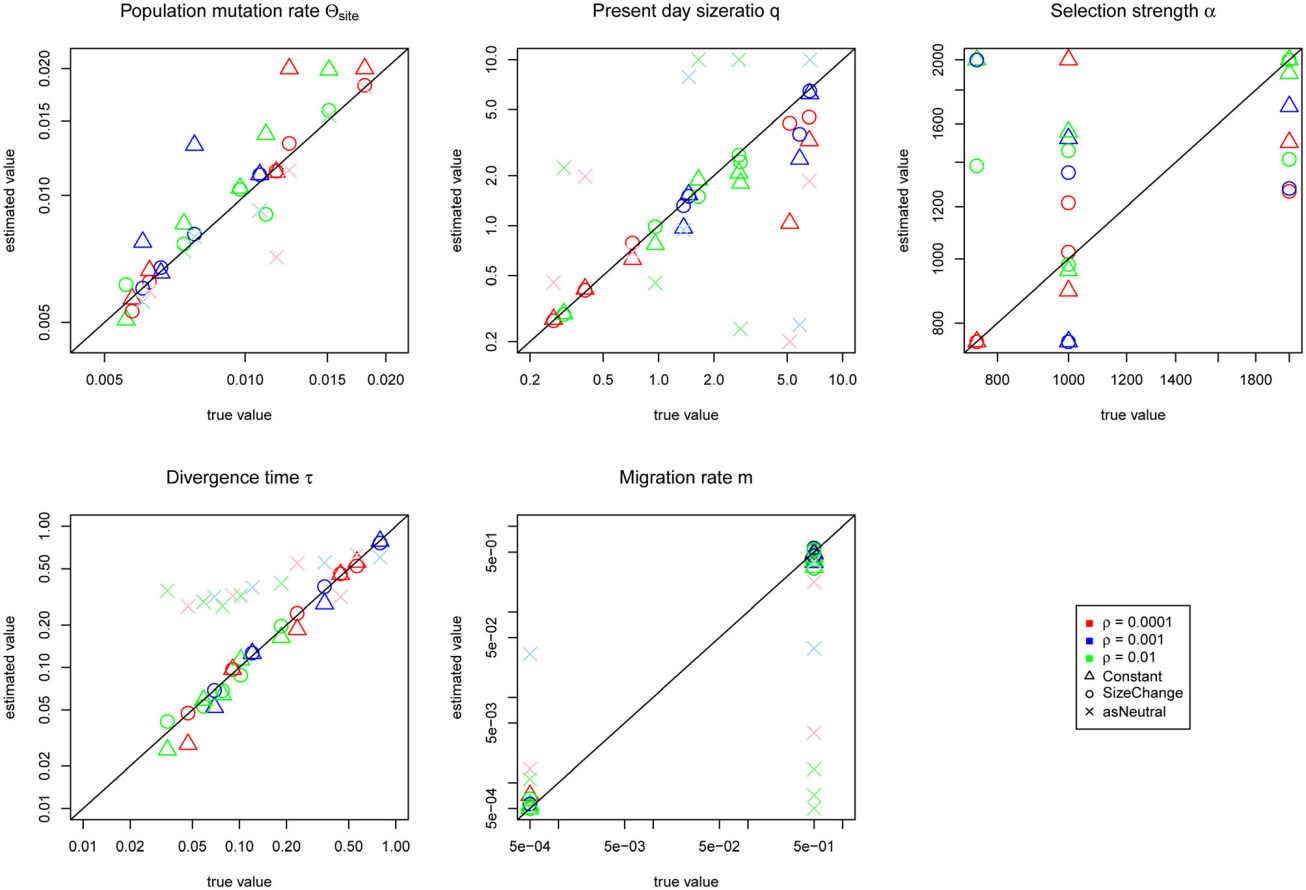
**Jaatha results of the simulation study under the complete sweep scenario for the *Constant* (Δ) and the *SizeChange* (o) model**. Data sets are color-coded according to their recombination rates per site of 10^-4^ (red), 10^-3^ (blue), and 10^-2^ (green). The results are plotted in the parameter ranges that were evaluated in Jaatha. The true value of the data sets produced with no migration were set manually to the lowest value of the parameter range. The demographic parameters are estimated accurately, however *α* is not. The divergence time of both populations (which also coincides with the timing of the selected allele and the migration rates) can be accurately inferred. θ, *q*, and α estimates lose precision compared to the incomplete sweep scenario. Incorrectly assuming neutrality (as Neutral for the *SizeChange* model) causes severe biases: overestimation of divergence times (always bigger than 0.2) and in most cases underestimation of migration rates, and associated mis-inference of the population size of *P_2_* (the population in which the selected allele arose). The recombination rate did not have any effect on the accuracy of the estimates.

When we conditioned on the selected allele being fixed (i.e., representing a complete sweep), all demographic parameters were estimated less accurately with the exception of the migration rate (**Figure 4**). In particular, the *Constant* model resulted in over-estimates of θ. Migration rates, however, were estimated with greater accuracy (cp. **Figures 3** and **4**). Unlike in the cases of incomplete sweeps, if we incorrectly assumed neutrality the estimates revealed severe biases, consistent with the results of Crisci et al. (2013). Divergence times were always estimated to be larger than 0.2 and migration rates were generally underestimated. Similarly poor results were obtained when we analyzed the complete sweep data sets with an incomplete sweep model (Supplementary Figure S4).

Applying this developed approach to the population of HCMV sampled from two compartments of a single individual (as within-host compartments have been demonstrated to diverge rapidly, see Renzette et al., 2013), indeed demonstrates an important role for both selective and demographic processes in shaping patterns of variation in this population. The model including selection gave a relatively improved fit versus its neutral counterpart (logL = -48 vs. -74). Most notably, the size of the population in the urine compartment is inferred to be much larger under a selection model (*q* = 39.56 relative to the ancestral size, vs. 0.51) and the time of the split of the urine and plasma compartments is also more recent under a selection model relative to a model assuming neutrality (*τ_s_* = 0.069 vs. 1.89 in units of *Ne* generations). The timing of selection is inferred to begin shortly after the colonization of the urine compartment (*τ_a_* = 0.054; in good correspondence with the estimate of the urine and plasma population divergence time above) and the average strength of selection is inferred as 2*Nes* = 4093 (resulting in an estimated *s* = 0.006 for our estimated *Ne*). Additionally, the size of the initial infection bottleneck of the fetus is estimated to be similarly severe (*u* = 0.09 of the ancestral size), and compartmentalization was estimated to occur quickly after the initial infection (*τ_b_* = 0.07).

## Discussion

Recent progress has been made in drawing joint inference of selection and demography from multiple time-point datasets, where inference is made from the trajectory of mutations through time, rather than from the site frequency spectrum (see Foll et al., 2014, 2015; also the recent review of Bank et al., 2014b). However, the great majority of datasets available are, and will likely remain, of the single time-point variety. Given the demonstrated biases introduced by assumptions of neutrality and equilibrium population histories in current estimators, and as no method currently exists to co-estimate selective and demographic parameters for single time-point data, we here seek to develop such an approach based on the JSFS.

Although the JSFS does not appear to have enough power to estimate selection strength precisely, it does distinguish between neutral and selected data sets, and even small (i.e., <100) and large (i.e., >> 100) alpha values. More significantly, we infer a subset of demographic parameters accurately under both scenarios (**Figures 3** and **4**). For completely neutral data sets, migration rates remain very difficult to estimate accurately as has been previously described (Naduvilezhath et al., 2011; Tellier et al., 2011). Including selection actually improves migration rate estimates under both the incomplete and the complete sweep scenarios as it becomes simpler to differentiate migrant alleles when a selected allele is introduced in one population only. This is supported by the fact that in the incomplete sweep scenario the higher the average frequency of the selected allele, the better the migration estimate obtained (**Figure 3**).

Further, with increasing growth rates, different selection strengths give rise to similar patterns of polymorphism (**Figure 2**), and thus it becomes increasingly difficult to estimate the correct present day population sizes (**Figure 4**). Particularly for the *Constant* model (with increasing true size ratios), size ratios are underestimated which in turn results in overestimation of the population mutation rate θ. In summary, in populations of changing size it is difficult to distinguish amongst positive selection coefficients, but it *is* possible to distinguish between neutral and positively selected sites.

Finally, if one incorrectly assumes neutrality, effects are comparatively minor under the incomplete sweep model. However, in the complete sweep model, estimation of demographic parameters became drastically biased under this assumption, as expected. Strikingly, in both sweep models the number of migrants (i.e., a product of present day size ratio *q* and migration rate *m*) is estimated correctly (see, e.g., three colered crosses of the neutral estimations in **Figure 3**), which is likely due to the easy distinction between individuals of both populations. Since migration rates are mostly underestimated in order to account for the observed differences between both populations, Jaatha responds by increasing the divergence times between the populations (see the overestimation particularly for low divergence times in **Figure 4**). Relatedly, Messer and Petrov (2013) report in their single-population simulations that methods inferring demography based on synonymous sites as a first step to detect selection, incorrectly estimate past population expansions. In our isolation-with-migration model, we similarly observe mis-estimation when the effects of selection are ignored, seen not only with population expansions but also contractions (**Figure 4**).

As an application of this approach, we jointly estimate the demographic and adaptive history of a population of HCMV within a single patient. With previous results suggesting both a strongly non-equilibrium history as well as pervasive positive selection during colonization, jointly estimating these parameters is of particular relevance. Firstly, we find that incorporating a model with selection into estimation of the demographic history at these chosen regions results in a better overall fit to the data. Comparing with the demographic estimates based on putatively neutral regions presented in Renzette et al. (2013), we find 1 a stronger estimated growth rate in the urine populations (40 times larger than the ancestral population, as opposed to 10 times – a result that is consistent with measured viral loads, and 2) a more recent split time between the plasma and urine population (0.054, compared to 0.53) – suggesting that compartmentalization may occur later in pre-natal infections than previously thought, as the inclusion of recurrent positive selection allows fixed differences to accumulate more rapidly between compartments relative to a neutral model. Additionally, the strength of selection acting in the plasma population is estimated to be strong, with mean 2*Ns* = 4093. However, there is at least one important similarity between the models – namely, the timing of the initial bottleneck representing fetal infection.

Thus, while this study demonstrates the utility of the JSFS for achieving joint selective and demographic estimation, it also identifies a number of notable limitations in parameter inference. In addition, a specific model is considered here in which multiple beneficial mutations arise at the time of the founding of a novel habitat or following an environmental shift. Considering the performance of this approach for models of selection on standing variation rather than *de novo* mutations, and for models of multiple competing beneficial mutations within a single locus, is worthy of future study. In addition, future improvements may be expected from the inclusion of additional SS outside of the JSFS, particularly including haplotype or linkage information, and additional software improvements will be valuable as this approach remains highly computationally intensive. Nonetheless, this work represents an important first step toward limiting the commonly made equilibrium assumptions in selection inference, and neutral assumptions in demographic inference, and suggests that future method development in this direction may indeed be fruitful.

## Author Contributions

JJ and LM designed the research and wrote the manuscript. LM conducted the simulation study and analyses.

## Conflict of Interest Statement

The authors declare that the research was conducted in the absence of any commercial or financial relationships that could be construed as a potential conflict of interest.
