# Supplementary material for: Evaluating the ability of the pairwise joint site frequency spectrum to co-estimate selection and demography
Source: Front Genet. 2015 Aug 17;6:268. doi: 10.3389/fgene.2015.00268 (PMC4538300; doi:10.3389/fgene.2015.00268)
Supplement: Supplementary file 1 [file Image_1.PDF]

SUPPLEMENTARY INFORMATION FOR

Evaluating the ability of the joint site frequency spectrum to co-estimate selection and demography

Lisha A. Mathew & Jeffrey D. Jensen

July 7, 2015

Corresponding Author: Jeffrey D. Jensen

EPFL

SV IBI UPJENSEN

Station 15

1015 Lausanne

Switzerland

+41 (0)21 69 38358

jeffrey.jensen@epfl.ch

Running Head: Co-estimation of demography and selection

Key Words: joint site frequency spectrum, joint estimation, selection and demography, single hitchhiking model, selection strength

A1. Parameter ranges for simulations and application, msms commands, and Jaatha 2.0 settings

For the incomplete sweep scenario:

The datasets were simulated with 100 loci and 25 samples per population and fixed $N_e = 10000$. The recombination rate ρ (per locus with locus length 1000 bp) was drawn uniformly from the given parameter range for half of the datasets and for the other half recombination rate was set to zero. In two randomly chosen datasets the migration rate was set to zero. The following parameters are population size scaled (according to Hudson, 2002; Ewing & Hermisson, 2010): θ, ρ, m, all time-related parameters τ_x, and α the selection strength. Given the parameter of the present day size ratio q and τ, the exponential growth rate g of population 2 can range from -40.5 to 300. The other parameters were chosen uniformly from the following ranges after log transformation:

population-scaled mutation rate θ **per locus** $\in [5, 20]$
recombination rate ρ $\in [1, 20]$
size ratios q $\in [0.01, 6]$
migration rate m $\in [0, 0.0005, 0.6]$
divergence time τ $\in [0.01, 16]$
selection strength α $\in [1 - 1000]$

msms command line (Ewing & Hermisson, 2010):

msms 50 100 -N 10000 -t θ -r ρ 1000 -I 2 25 25 -m 1 2 m -m 2 1 m -n 2 q -eN τ (1+u) -ej τ 2 1 -Sp 0.5 -SA α -SI τ 2 0 $1/(u{*}N)$ -SFC

with $u = 0.3$ for the *SizeChange* model and $u = q$ for the *Constant* model. Size change in the *SizeChange* model was added with -g 2 $\frac{log(\frac{q}{u})}{\tau}$.

For the complete sweep scenario:

The same msms command line as in the incomplete scenario were also used but a locus was only retained in the dataset if the selected site was fixed. Given the parameter of the present day size ratio q and τ, the exponential growth rate g of population 2 can range from -40.5 to 350. The following parameter ranges were different compared to the incomplete sweep scenario with ρ, α, and m no longer being drawn randomly:

recombination rate ρ**:** $0.1, 1, 10$

size ratios $q \in [0.2, 10]$

divergence time $\tau \in [0.01, 1]$

selection strength α**:** $750, 1000, 2000$

migration rate m**:** $0, 0.5$

For the hCMV application:

The demographic model and the fixed parameters were chosen based on the neutral demography estimations of Renzette *et al.* (2013). A neutral and a selection model were run on 15 calculated regions based on the significant population branch statistic results of Renzette *et al.* (2013).

present day size ratios $q \in [0.2, 500]$

size ratios at split time $s \in [0.001, 7]$

size ratios at bottleneck $u \in [0.001, 2]$

divergence time $\tau_S \in [0.01, 7]$

duration of bottleneck $\tau_B \in [0.0001, 1.5]$

start of selection $\tau_a \in [0.01, 0.2]$

selection strength $\alpha \in [500 - 5000]$

msms command line (Ewing & Hermisson, 2010):

for neutral model msms 30 15 -t θ -I 2 15 15 0 -n 2 q -g 2 (log(q/s))/τ_S -ej τ_S 2 1 -eN τ_S (1+s) -eG τ_S (log((1+s)/u))/τ_B -eN (τ_B+τ_S) 24.3 -r 20 1000

for selection model msms 30 15 -N 1275-t θ -I 2 15 15 0 -n 2 q -g 2 (log(q/s))/τ_S -ej τ_S 2 1 -eN τ_S (1+s) -eG τ_S (log((1+s)/u))/τ_B -eN (τ_B+τ_S) 24.3 -r 20 1000 -Sp 0.5 -SA α -SFC -SI min(τ_S,τ_a) 2 1/1275 0

Jaatha 2.0 settings for all analyses (Mathew et al.*, 2013):*

Following the nomenclature in Mathew *et al.* (2013): $k = 3$, $s_{main} = 200$, $w = 0.9$, $\epsilon = 0.9$, $r = 0.05$, $s_{ini} = 200$, $n_{RP} = 10$, $n_{SS} = 23$, ext_θ =TRUE, $M_{ini} = M_{main}$ =infinite sites model, $s_{final} = 500$, $scale = 1$, and $t_{max} = 200$ for the incomplete sweep scenario and $t_{max} = 150$ for the complete sweep scenario.

References

Ewing G, Hermisson J (2010) MSMS: a coalescent simulation program including recombination, demographic structure and selection at a single locus. *Bioinformatics*, **26**, 2064–2065.

Hudson RR (2002) Generating samples under a Wright-Fisher neutral model of genetic variation. *Bioinformatics*, **18**, 337–338.

Mathew LA, Staab PR, Rose LE, Metzler D (2013) Why to account for finite sites in population genetic studies and how to do this with Jaatha 2.0. *Ecol Evol*, **3**, 3647–3662.

Renzette N, Gibson L, Bhattacharjee B, *et al.* (2013) Rapid intrahost evolution of human cytomegalovirus is shaped by demography and positive selection. *PLoS Genet*, **9**, e1003735.

A2. Additional Figures

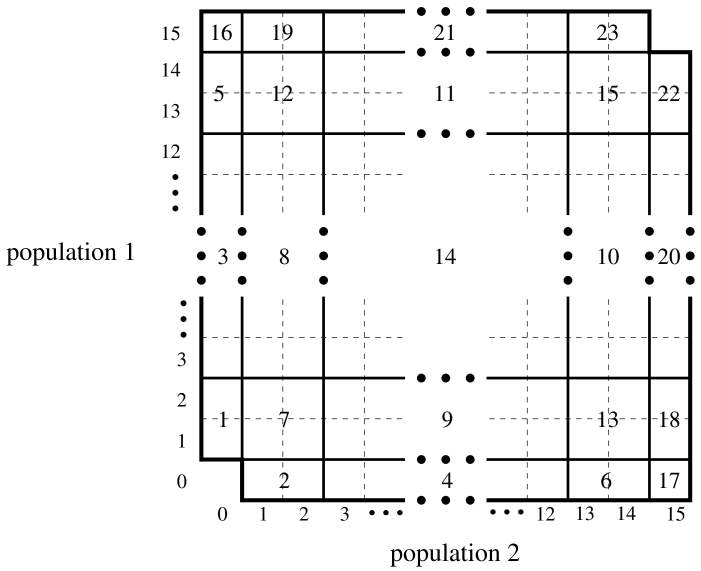

Figure S1.—: The joint site frequency spectrum of two populations is visualized with 15 individuals sampled from each population. Jaatha's default set of 23 summary statistics is being calculated by binning the different indicated frequency classes.

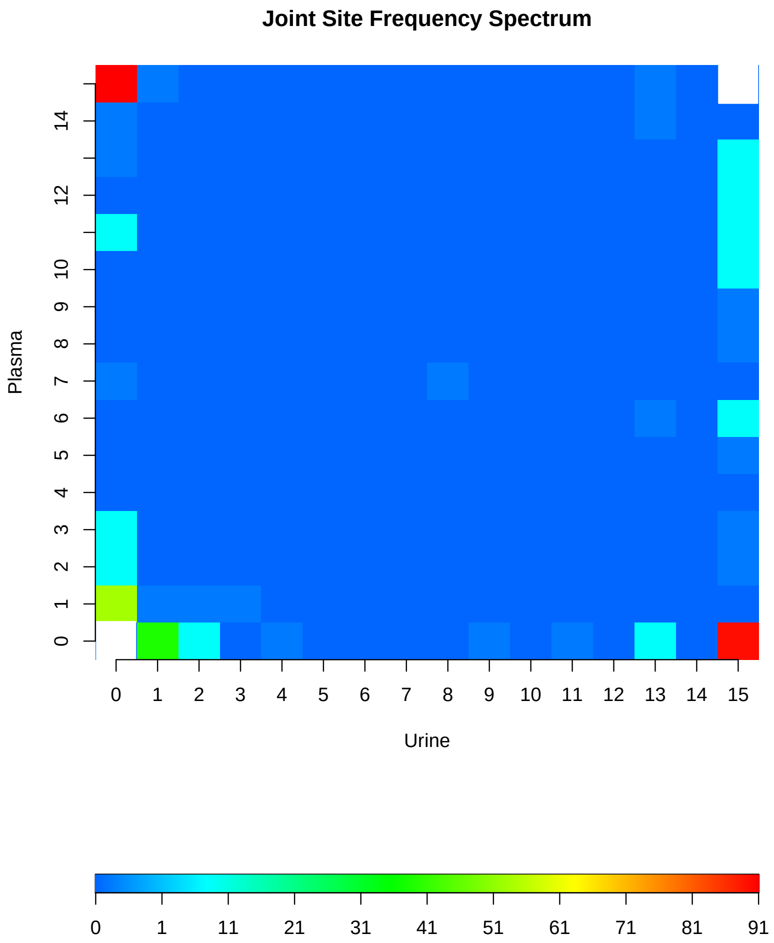

Figure S2.—: The calculated JSFS for 15 regions of the application dataset of human cytomegalovirus (based on published data by Renzette *et al.* (2013)). The population sample size was set to 15 because the minimal coverage criterion for a site was set to that value. Positions with higher coverages were scaled down to 15.

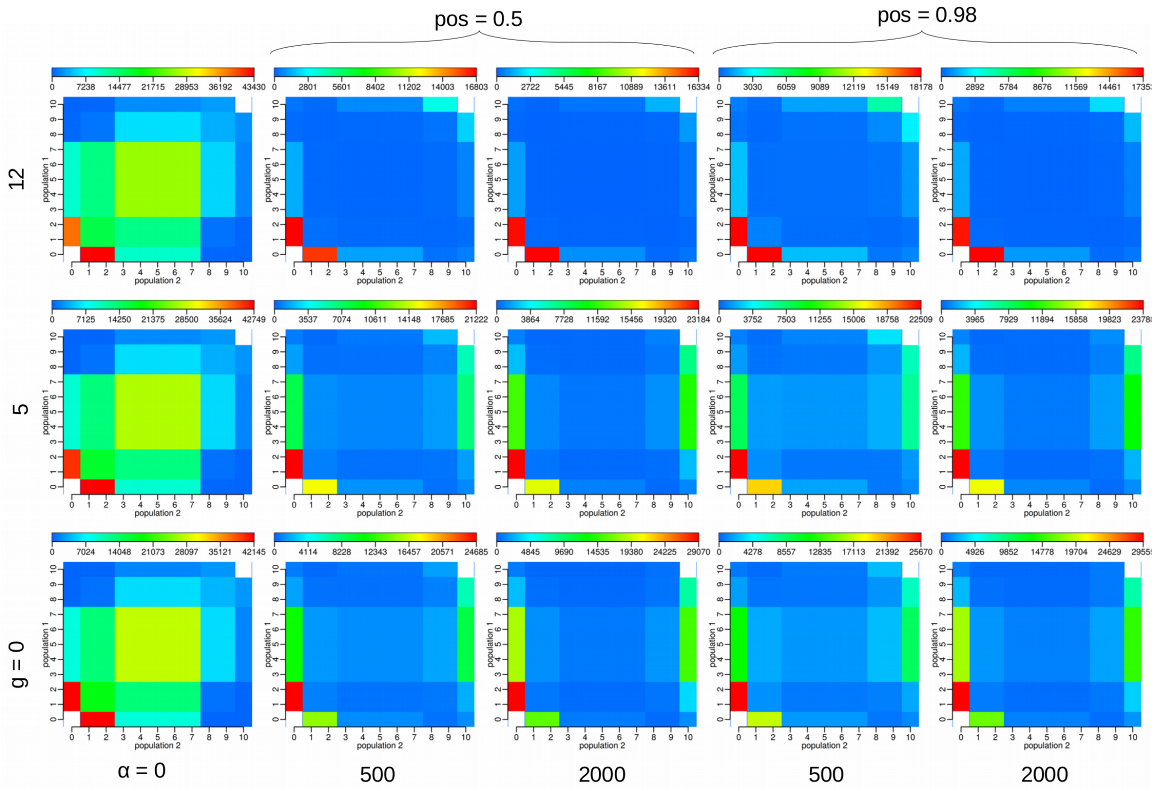

Figure S3.—: We visualized the average values of the 23 summary statistics (SS) used in this article with three differing parameters under the *SizeChange* model under a complete sweep scenario: selection strength $\alpha = 2N_e s$, the exponential growth rate of the second population g in which also the selected allele arises, and the position of the selected allele pos. For each plot we ran 100 replicates of each 100 loci of 100 kb in length with 10 samples from each population. The other simulation parameters were fixed to the following values: τ=0.05, N_e=1000, θ_{site}=0.004, m=0.2, and recombination rate per site = $1.64 \cdot 10^{-4}$. The distinction between the neutral and selected cases is clearly visible which is due to the decrease in polymorphisms in the selected case. The higher the growth rate g gets, the closer in number and more similar the SS values become. The SS show little differences for the different positionings of the selected allele but larger ones than in the case when we simulated only 1kb loci (cp. Fig. 2).

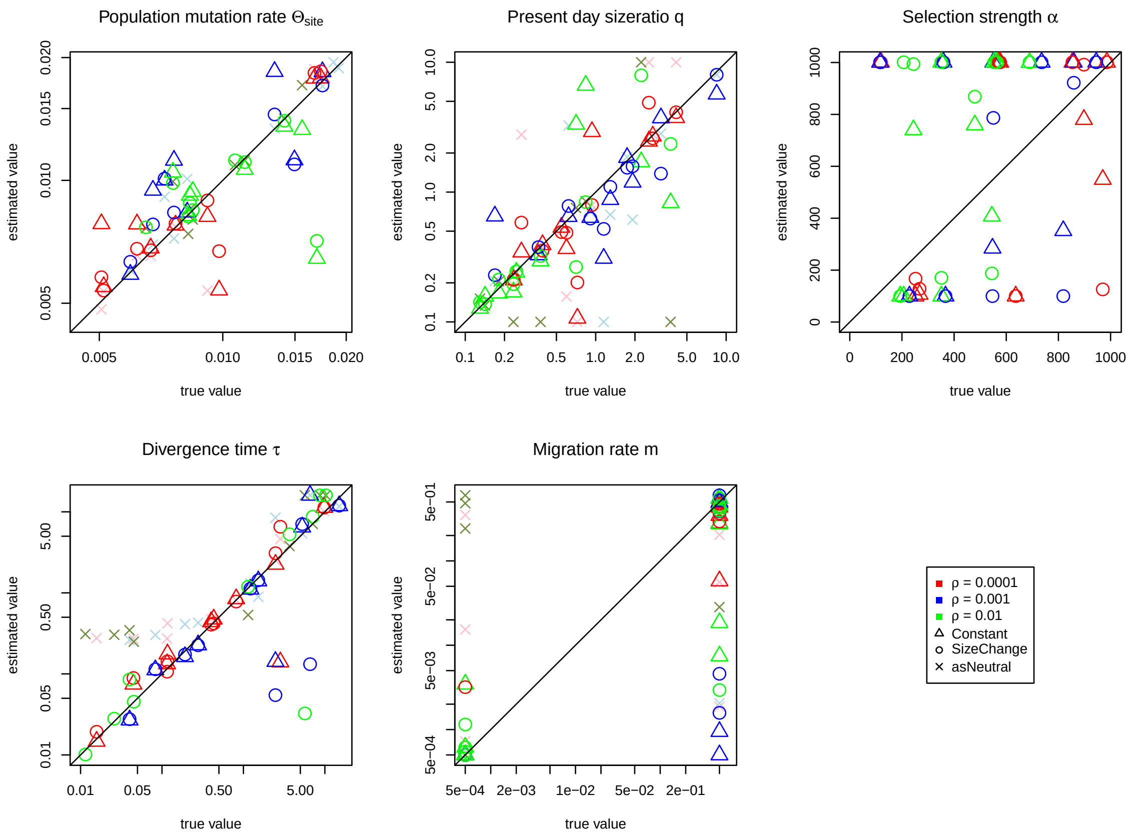

Figure S4.—: Result of Jaatha runs on datasets simulated under the *Constant* and *SizeChange* model with a complete sweep, but analyzed with an incomplete sweep model (or the neutral model (x)). Except for divergence times, estimations lose accuracy. Incorrectly assuming a neutral model results in most cases in even greater mis-estimation. Especially low divergence times cannot be recovered and are estimated always at least to be 0.2. The corresponding migration rates are overestimated.
